# Molecular detection of parasites and host preference in wild-caught *Culicoides* biting midges (Diptera: Ceratopogonidae) in Chiang Mai and Nakhon Si Thammarat Provinces, Thailand

**DOI:** 10.1051/parasite/2024082

**Published:** 2025-01-22

**Authors:** Baby Kyi Soe, Saowalak Kaewmee, Chonlada Mano, Urassaya Pattanawong, Nopporn Tipparawong, Padet Siriyasatien, Derek Gatherer, Michael D. Urbaniak, Paul A. Bates, Narissara Jariyapan

**Affiliations:** 1 Center of Excellence in Vector Biology and Vector-Borne Disease, Department of Parasitology, Faculty of Medicine, Chulalongkorn University Bangkok 10330 Thailand; 2 Molecular Biology of Malaria and Opportunistic Parasites Research Unit, Department of Parasitology, Faculty of Medicine, Chulalongkorn University Bangkok 10330 Thailand; 3 Department of Pathology, King Chulalongkorn Memorial Hospital Bangkok 10330 Thailand; 4 Division of Biomedical and Life Sciences, Faculty of Health and Medicine, Lancaster University Lancaster LA1 4YG United Kingdom

**Keywords:** *Culicoides*, Midge, *Leucocytozoon*, *Plasmodium*, Parasites, Thailand

## Abstract

*Culicoides* biting midges (Diptera: Ceratopogonidae) have been reported as potential vectors for haemoparasites. Information about host-vector-parasite specificity is required to confirm their status. Here, molecular detection of haemosporidians, *Leishmania*, trypanosomatids, and filarial nematodes in biting midges was conducted to understand their potential role as vectors, and their host preference was determined. Wild-caught biting midges were collected from six different localities of Chiang Mai and Nakhon Si Thammarat provinces, Thailand. A total of 6,578 individual *Culicoides* (170 males, 6,408 females) comprising 15 species of six sub-genera and two groups were collected. Also, 738 parous females and 29 engorged females were examined for parasites and host blood meals, respectively. *Culicoides arakawae*, *C*. *mahasarakhamense*, *C*. *peregrinus* and *C*. *innoxius* in Chiang Mai province, and *C*. *innoxius* and *C*. *peregrinus* in Nakhon Si Thammarat province were the most dominant species. *Leucocytozoon* spp., *Leucocytozoon caulleryi* and *Plasmodium juxtanucleare* were identified in five *Culicoides* species including *C*. *mahasarakhamense*, *C*. *arakawae*, *C*. *oxystoma*, *C*. *fulvus*, and *C*. *guttifer*. This study is the first record of *L. caulleryi* in the biting midge *C*. *arakawae* in Thailand. Blood meal analysis revealed that *Culicoides* primarily fed on cattle (17/29, 58.6%), followed by chickens (10/29, 34.5%), and humans (2/29, 6.9%). Our findings confirmed the existence of several *Culicoides* species in Thailand, which might be potential vectors for transmission of haemosporidians (*Leucocytozoon* and *Plasmodium*). Information from host blood meal analyses underlined their preference for large mammals, followed by domestic chickens. More anthropophilic *Culicoides* species remain to be discovered.

## Abstract (fr)

Les culicoïdes (Diptera : Ceratopogonidae) ont été signalés comme vecteurs potentiels d’hémoparasites. Des informations sur la spécificité hôte-vecteur-parasites sont nécessaires pour confirmer leur statut. Ici, la détection moléculaire des hémosporidies, des leishmanies, des trypanosomatidés et des nématodes filaires chez les culicoïdes a été réalisée pour comprendre leur rôle potentiel en tant que vecteurs, et leur préférence d’hôte a été déterminée. Les culicoïdes capturés dans la nature ont été collectés dans six localités différentes des provinces de Chiang Mai et Nakhon Si Thammarat, en Thaïlande. Un total de 6 578 culicoïdes (170 mâles, 6 408 femelles), comprenant 15 espèces de six sous-genres et deux groupes, ont été collectés. De plus, 738 femelles pares et 29 femelles gorgées ont été examinées respectivement pour détecter des parasites et l’hôte de leur repas sanguin. Les espèces les plus dominantes étaient *Culicoides arakawae*, *C. mahasarakhamense*, *C. peregrinus* et *C. innoxius* dans la province de Chiang Mai, et *C. innoxius* et *C. peregrinus* dans la province de Nakhon Si Thammarat. *Leucocytozoon* spp., *Leucocytozoon caulleryi* et *Plasmodium juxtanucleare* ont été identifiés chez cinq espèces de *Culicoides*, dont *C. mahasarakhamense*, *C. arakawae*, *C. oxystoma*, *C. fulvus* et *C. guttifer*. Cette étude est la première observation de *L. caulleryi* chez le culicoïde *C. arakawae* en Thaïlande. L’analyse des repas sanguins a révélé que les culicoïdes se nourrissaient principalement sur les bovins (17/29, 58,6 %), suivis des poulets (10/29, 34,5 %) et des humains (2/29, 6,9 %). Nos résultats ont confirmé l’existence de plusieurs espèces de *Culicoides* en Thaïlande, qui pourraient être des vecteurs potentiels de transmission d’hémosporidies (*Leucocytozoon* et *Plasmodium*). Les informations obtenues par analyse des repas de sang des hôtes ont souligné leur préférence pour les grands mammifères suivis par les poulets domestiques. D’autres espèces de culicoïdes anthropophiles restent à découvrir.

## Introduction

Haematophagous arthropods represent a crucial threat due to their role in transmitting life-threatening diseases to wild vertebrates [5]. Nowadays, vector-borne diseases transmitted by the blood-feeding arthropods are of significant global public health concern due to their widespread impact, morbidity, and mortality [30]. Besides human welfare considerations, the arthropod vectors can pose significant challenges for the livestock industry, potentially resulting in significant economic losses [48]. *Culicoides* biting midges (Diptera: Ceratopogonidae), the most common small blood-feeding insects, are known to transmit viruses, such as Bluetongue virus and Oropouche virus [31, 35].

Regarding parasites, *Culicoides* biting midges are considered to transmit avian haemosporidians, such as *Leucocytozoon* spp., *Plasmodium* spp., and *Haemoproteus* spp., which are responsible for causing diseases in birds [39, 61]. According to previous research, *Culicoides* biting midges have been proposed as potential vectors of specific pathogens, with some being linked to the transmission of zoonotic diseases, *i.e.*, leishmaniasis caused by some members of *Leishmania* parasites in the subgenus *Mundinia*. For example, experimental infection by *Leishmania* (*M.*) *orientalis* in *C. sonorensis* has been demonstrated by Chanmol *et al.* [8], and the capability of biting midges *C*. *sonorensis* to establish infection and potential transmission of *Leishmania* (*M.*) parasites has been reported by Becvar *et al.* [3]. Also, *C*. *mahasarakhamense* has been reported to contain *L*. *martiniquensis* DNA [54]. Next, *Leishmania* (*M.*) *martiniquensis* DNA has been investigated in several *Culicoides* species, such as *C*. *peregrinus*, *C*. *oxystoma*, *C*. *mahasarakhamense*, *C*. *huffi*, *C*. *fordae*, and *C*. *fulvus* in southern Thailand [50]. In addition, a report on natural infection of *C*. *peregrinus* by *L*. *martiniquensis* has provided evidence to support the notion of biting midges as a potential vector of leishmaniasis, and two *C*. *peregrinus* samples were found to be coinfected with *L*. *martiniquensis* and *Crithidia* sp. [22]. Recently, *Leishmania* sp. DNA was detected from six *Culicoides* spp.: *C*. *mahasarakhamense*, *C*. *guttifer*, *C*. (*Trithecoides*) sp., *C*. *jacobsoni*, *C*. *oxystoma*, and *C*. *orientalis*, indicating their important role in the transmission of this zoonotic pathogen [1]. For filarial nematodes, natural infections by *Mansonella perstans*, *M*. *ozzardi*, and *M*. *streptocerca* in *Culicoides* spp., which are responsible for mansonellosis in humans, have been recorded [35]. Also, DNA of Onchocercidae gen. sp. has been detected in *C*. *crepuscularis* [31], *C*. *mahasarakhamense* [40], and *M*. *perstans* in *C*. *milnei* [10] through molecular studies, suggesting the potential for these *Culicoides* species to be a vector of this filarial parasite.

Studies on the parasite infection status and host preference of *Culicoides* biting midges have become critical to understanding the transmission pathways of zoonotic vector-borne diseases. Determining blood meal remnants in arthropods may outline a comprehensive interaction between ectoparasites and hosts, and their efficiency of pathogen transmission [47]. *Culicoides* biting midges feed on a wide range of mammals and birds, depending on the abundance and accessibility of hosts [28]. In a previous study, avian DNA was detected in *C*. *mahasarakhamense* and *C*. *huffi* [54]*.* In addition, Sunantaraporn *et al.* [53] reported that not only cow, dog, pig, or avian DNA was found in each engorged midge collected in their study, but also the most prevalent blood meal pattern, mixed host blood DNA (cow and avian), was identified in *Culicoides* spp. of the subgenus *Trithecoides*, *C*. *innoxius*, *C*. *peregrinus*, *C*. *shortti*, *C*. *fulvus*, *C*. *insignipennis*, *C*. *jacobsoni*, and *C*. *gemellus*. Since the presence of suitable hosts may influence the abundance and distribution of the midges, understanding their habits in endemic areas of diseases, particularly host preference, is beneficial. Thailand is well known for its diverse environment, featuring a variety of ecosystems, such as wetlands, coastal areas, mountainous regions, tropical rainforests, and agricultural lands, which in turn represent a wide range of climate zones. Most farmers in Thailand practice mixed-livestock farming in which cattle, goats, chickens, and dogs are raised, living alongside people in the same residential areas. The occurrence of human blood DNA in several *Culicoides* species has been reported from several studies in different countries [6, 14, 23–26, 46, 49, 57]. Nonetheless, *Culicoides* are described as opportunistic feeders, and host feeding behavior may be strongly influenced by host availability [35]. For example, Slama *et al.* [49] have acknowledged that a high percentage of *C. imicola* blood feeding on humans in central Tunisia can most probably be attributed to the location of the traps in the direct vicinity of human habitats. However, opportunistic host feeding may facilitate pathogen transfer between wild and domestic hosts or even to humans [35].

Further investigation on the host preference of various species of *Culicoides* biting midges is needed. The aims of the present study, therefore, are to (i) investigate the most dominant biting midge species in selected mixed livestock farming areas in Chiang Mai and Nakhon Si Thammarat provinces, Thailand, (ii) identify blood parasites in the wild-caught biting midges, and (iii) determine host preference *via* blood meal analysis of the midges. These findings may contribute to an understanding of the role of *Culicoides* biting midges in the transmission of parasitic infections. Importantly, this knowledge could offer a roadmap to predicting possible vector-parasite specificity, planning future research on prevention, and improving existing control strategies.

## Materials and methods

### Ethics statement

The design and methodology of this research was approved by the animal research ethics committee of Chulalongkorn University Animal Care and Use Protocol (CU-ACUP) under COA No. 037/2566, Faculty of Medicine, Chulalongkorn University, Bangkok, Thailand.

### Study period, location, and insect trapping

**Figure 1:**
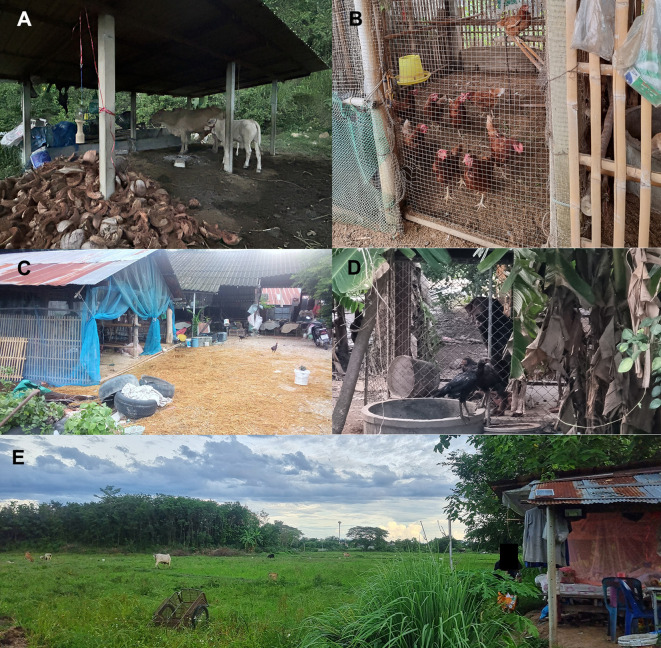
Examples of sample collection sites. A: Cattle pen nearby forest; B–D: Mixed-livestock farming with cattle, chickens, and dogs nearby farm owners’ houses; E: Semi-intensive farming practice with cattle grazed nearby pasture.

**Figure S1:**
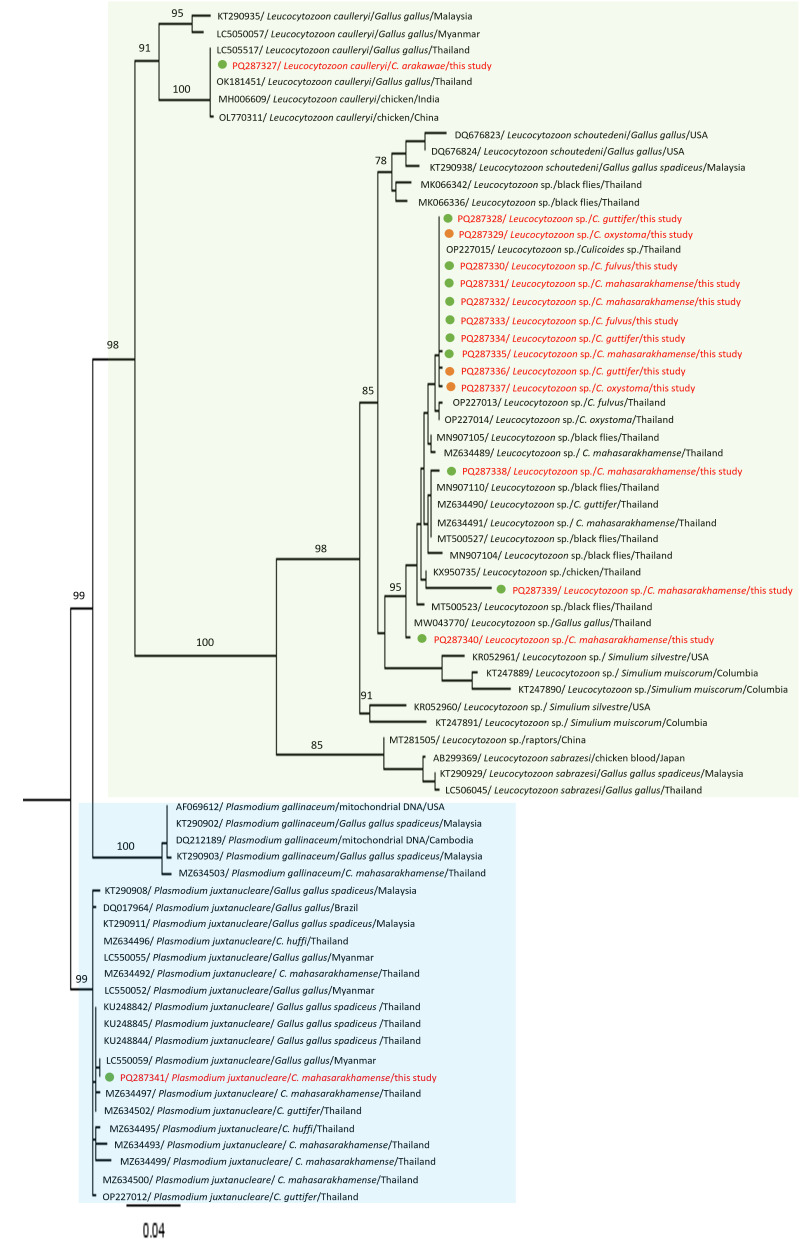
Map showing the 6 sampling sites with geographical boundaries: 3 sites in Chiang Mai; San Sai, Doi Saket, and Hang Dong, and 3 sites in Nakhon Si Thammarat; Nopphitam, Tha Sala, and Na bon. The map was drawn using QGIS version 3.34.

The present study was carried out between February 2024 and July 2024. In this study, three districts (San Sai, Doi Saket, and Hang Dong) in Chiang Mai province were involved as the northern sample collection area, while three districts (Na Bon, Nopphitam, and Tha Sala) in Nakhon Si Thammarat province were included as the southern sample collection area (Table 1, Fig. S1). The two study areas (northern and southern parts of Thailand) are in different climatic zones: Chiang Mai is characterized by a tropical savannah climate, and Nakhon Si Thammarat has a tropical rainforest climate. Annual rainfall is 1,185 mm and 2,381 mm in Chiang Mai and Nakhon Si Thammarat, respectively (https://en.climate-data.org/asia/thailand/). Farming practices in the study areas are categorized as mixed-livestock farming systems, in which people live near their farms. At each location, sampling took place over three days. A total of six trapping localities from the two study areas were selected, according to urban natural environments at the sample collection sites. Following verbal permission from the house owner, insects were collected using light traps (25 W bulb) with ultraviolet (UV) light, as shown in Figure 1. Briefly, at each site, a total of 6 light traps were set up approximately 1.5 m above ground level for three consecutive nights/month. The operating hours of the traps were from 6 pm to 6 am. The collected biting midges were subsequently sent to the laboratory of the Center of Excellence in Vector Biology and Vector-Borne Disease Research Unit, Department of Parasitology, Faculty of Medicine, Chulalongkorn University.

**Table 1:** Details of collection sites in Chiang Mai and Nakhon Si Thammarat provinces, Thailand.

Location	Coordinates	Altitude (m)	Animals (number)			Distance from house (m)
			Cattle	Chicken	Dog	
Chiang Mai						
San Sai	18°53′29.0″N 99°06′46.4″E	1,123	15	>60	2	10
Doi Saket	18°49′23.8″N 99°05′09.1″E	1,107	10	>50	2	10
Hang Dong	18°42′39.6″N 98°54′13.6″E	1,126	12	>50	2	10
Nakhon Si Thammarat						
Na Bon	8°15′45.7″N 99°37′55.6″E	919	10	>20	1	15
Nopphitam	8°47′19.5″N 99°42′33.5″E	958	6	0	2	30
Tha Sala	8°36′35″N 99°51′45″E	856	15	>20	1	20

### Morphological identification, DNA extraction, and molecular identification of midges

Identification of different species of *Culicoides* was performed. First, males were separated from females. Thereafter, species identification of female specimens was conducted using a taxonomic key, based on morphological characteristics such as head features (including the palp and antenna) and wing patterns [59]. Briefly, the wings of biting midges were examined, and the following information was recorded: size, length, either dark or pale spots on the entire wing with their particular locations, wing color, and situation of the microtrichia. For each species, classification of female insects was carried out based on their physiological stage: parous (empty abdomen with remnants of burgundy pigment), engorged (abdomen full of blood), gravid (abdomen containing eggs), and nulliparous (empty abdomen without blood remnants) [26]. The wings of representative insects were dissected, and images were taken. The remaining insect bodies were subjected to DNA extraction. Genomic DNA was extracted using a genomic DNA extraction kit (Thermo Fisher Scientific Inc., Waltham, MA, USA), according to the manufacturer’s instructions. In this study, 1–30 parous females from each species (depending on the number of collected parous females) were randomly selected and individually used for DNA extraction. To identify *Culicoides* species through molecular techniques, the primer pair targeting the mitochondrial cytochrome c oxidase subunit 1 gene (COI) was used [13], following the PCR protocol described by Harrup *et al.* [16].

### Molecular identification of *Leishmania* and trypanosomatids

For *Leishmania* spp., the internal transcribed spacer 1 (ITS1) [51] and 3′ untranslated region (3′UTR) of *Leishmania HSP70*-type I (*HSP70-I*) [19, 44] were used, with DNA sequencing for species identification. For *Trypanosoma* spp. and *Crithidia* spp., small subunit ribosomal RNA (SSU rRNA) gene was targeted for amplification with the primer pair reported by Noyes *et al.* [36], following the PCR protocol described by Srisuton *et al.* [52] and Kaewmee *et al.* [22].

### Molecular identification of *Leucocytozoon* spp., *Plasmodium* spp., and *Haemoproteus* spp.

In order to identify *Leucocytozoon* spp., *Plasmodium* spp., and *Haemoproteus* spp. DNA, the extracted genomic DNA samples were amplified by nested PCR (nPCR). Briefly, nPCR targeting the cytochrome *b* (cyt *b*) gene was conducted using the outer primer pairs described by Hellgren *et al.* [17]. For nested PCR, the inner primer pairs previously reported by Bensch *et al.* [4] were used to amplify *Plasmodium* and *Haemoproteus* species.

### Molecular identification of filarial nematodes

For filarial nematode identification, PCR amplification targeting the mitochondrial 12S ribosomal RNA (12S rRNA) gene was carried out using the primer pairs described by Morales-Hojas *et al.* [34], following the PCR protocol described by Pramual *et al.* [40].

### Blood-meal molecular analysis

Total genomic DNA was extracted from blood meal remnants from the 29 engorged female *Culicoides* using a DNA extraction and purification kit (Thermo Fisher Scientific Inc.), as per the manufacturer’s protocol. The extracted genomic DNA samples were stored at −20 °C until molecular identification. The universal vertebrate primers for amplification of the mitochondrial cytochrome *b* (cyt *b*) gene were used to identify host blood meal [42] with the following PCR cycling procedure, with some modifications: initial denaturation at 94 °C for 5 min, 35 cycles of denaturation at 94 °C for 1 min, annealing at 55 °C for 1 min, extension at 72 °C for 1 min, followed by final extension at 72 °C for 10 min, with a total volume of 25 μL reaction mixture. The list of the primers used in the present study is shown in Table 2.

**Table 2:** List of primers used for PCR amplification and sequencing.

Pathogen/Host	Primer	Oligonucleotide sequence (5′ to 3′)	Targeted gene	Product (bp)	References
*Culicoides* spp.	LCO1490	GGTCAACAAATCATAAAGATATTGG	COI	~ 658	[13]
	HCO2198	TAAACTTCAGG GTGACCAAAAAATCA			
*Leishmania* spp.	LeF	TCCGCCCGAAAGTTCACCGATA	ITS1	~ 379	[51]
	LeR	CCAAGTCATCCATCGCGACACG			
	70-IR-D	CCAAGGTCGAGGAGGTCGACTA	*HSP70-I*	~ 754	[44]
	70-IR-M	ACGGGTAGGGGGAGGAAAGA			
Trypanosomatids	Try927F	GAAACAAGAAACACGGGAG	SSU rRNA	~ 927	[36]
	Try927R	CTACTGGGCAGCTTGGA			
Haemosporidians	HaemNFI	CATATATTAAGAGAAITATGGAG	cyt *b*	~ 618	[17]
	HaemNR3	ATAGAAAGATAAGAAATACCATTC			
*Plasmodium* and *Haemoproteus*	HaemF2	ATGGTGCTTTCGATATATGCATG	cyt *b*	~ 480	[4]
	HaemR2	GCATTATCTGGAT GTGATAATGGT			
*Leucocytozoon*	HaemFL	ATGGTGTTTTAGATACTTACATT	cyt *b*	~ 478	[17]
	HaemR2L	CATTATCTGGATGAGATAATGGIGC			
Filarial nematodes	12SOvC.F	TCGGCTATGCGTTTTAATTTT	12S rRNA	~ 550	[34]
	12SOvB.R	CAACTTACGCCCCTTTAGGC			
Vertebrates	cyt bb1	CCATCMAAC ATYTCADCATGATGAAA	cyt *b*	~ 350	[42]
	cyt bb2	GCHCCTCAGAATGAYATTTGKCCTCA			

### DNA sequencing

The amplified products were purified using a GeneJET PCR Purification kit (Thermo Fisher Scientific Inc.) and sent for Sanger sequencing at the sequencing service of Macrogen Inc., Seoul, South Korea. Here, we sequenced DNA from (i) insects that were found to be positive for parasites by molecular methods, and (ii) engorged female insects only.

### Phylogenetic analyses

The resulting nucleotide sequences of the mitochondrial cytochrome *b* (cyt *b*) gene of blood meal remnants, and cyt *b* genes of haemosporidian parasites (*Leucocytozoon* spp. and *Plasmodium* spp.) were checked using a BLASTn search tool (https://blast.ncbi.nlm.nih.gov/Blast.cgi). Reference sequences were retrieved from GenBank to conduct phylogenetic analyses in order to determine genetic similarity and diversity. Briefly, multiple sequence alignment was performed with Molecular Evolutionary Genetics Analysis (MEGA), software version 11, and the genetic inference was analyzed by the maximum likelihood method using IQ-TREE software https://iqtree.org/ [27, 45]. A 1,000 repetition bootstrapping test was used to evaluate the confidence of the branching pattern [11], in which the bootstrap values ≥70% were taken as an indication of support [18]. Evolutionary distances were computed using the code to find the best model. The phylogenetic tree was visualized using FigTree, version 1.4 [43].

## Results

### *Culicoides* species found in the mixed-livestock farming areas

**Figure 2:**
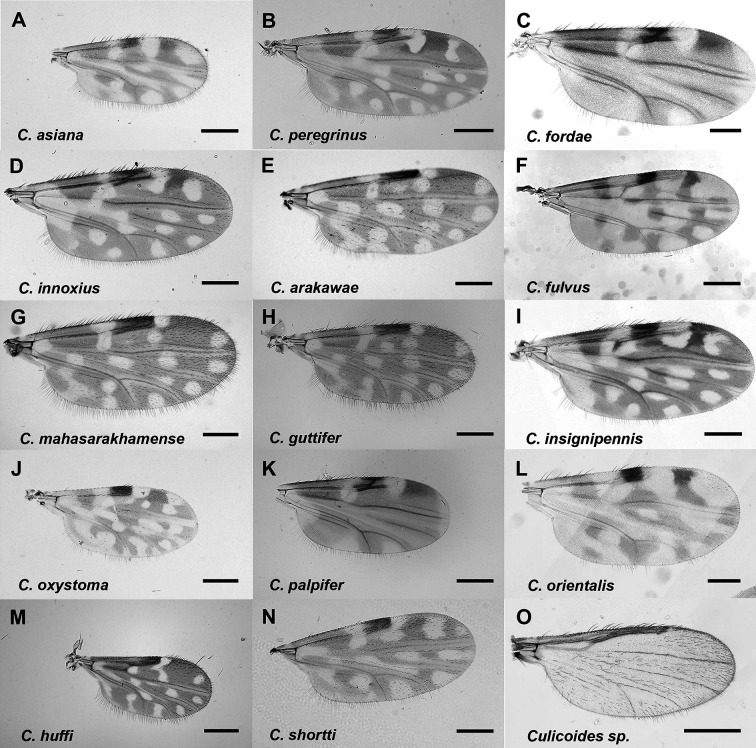
Characteristics of wing patterns of 15 *Culicoides* species identified from this study. Scale bar = 200 μm.

In the present study, a total of 6,578 *Culicoides* spp., 170 males and 6,408 females (5,275 parous, 1,092 nulliparous, 12 gravid, and 29 engorged) were collected, comprising 15 species of six subgenera (*Hoffmania*, *Meijerehelea*, *Remmia*, *Avaritia*, *Haemophoructus*, and *Trithecoides*), and two groups (Clavipalpis and Shortti). These species included *C*. *asiana* (2.6%), *C*. *innoxius* (20.5%), *C*. *mahasarakhamense* (13.2%), *C*. *arakawae* (15.3%), *C*. *peregrinus* (20.9%), *C*. *oxystoma* (7.6%), *C*. *palpifer* (4.4%), *C*. *huffi* (3.1%), *C*. *shortti* (2.5%), *C*. *guttifer* (2.9%), *C*. *fulvus* (2.8%), *C*. *orientalis* (1.1%), *C*. *fordae* (2.3%), *C*. *insignipennis* (0.2%), and *Culicoides* spp. (unknown) (0.6%) depending upon the characteristics of wing patterns (Table 3). Geographically, *C*. *arakawae* 18.7% (716/3829), *C*. *mahasarakhamense* 18.1% (692/3829), *C*. *peregrinus* 17.5% (672/3829), and *C*. *innoxius* 15.6% (599/3829) were the most dominant *Culicoides* spp. in Chiang Mai, while *C*. *innoxius* 27.3% (752/2749) and *C*. *peregrinus* 25.6% (705/2749) were the most dominant in Nakhon Si Thammarat; therefore, *C*. *peregrinus* and *C*. *innoxius* seemed to be widely spread in both the southern and northern parts of Thailand. Characteristics of wing patterns of 15 representative *Culicoides* species identified from the current study are shown in Figure 2.

**Table 3:** Total number of *Culicoides* species captured in Chiang Mai and Nakhon Si Thammarat provinces, Thailand, February–July 2024.

Sr. No.	*Culicoides* species	Number of biting midges												All Total
		Chiang Mai						Nakhon Si Thammarat						
		Male	Female				Total	Male	Female				Total	
			Parous	Engorged	Gravid	Nulliparous			Parous	Engorged	Gravid	Nulliparous		
1.	*C*. *asiana*	15	92	0	0	20	127	2	26	0	0	15	43	170
2.	*C*. *peregrinus*	22	537	3	2	108	672	12	605	4	2	82	705	1,377
3.	*C*. *innoxius*	21	481	3	1	93	599	19	647	3	2	81	752	1,351
4.	*C*. *arakawae*	15	561	3	0	137	716	6	226	0	0	56	288	1,004
5.	*C*. *mahasarakhamense*	18	581	4	0	89	692	3	137	0	0	34	174	866
6.	*C*. *oxystoma*	12	264	1	0	82	359	1	122	0	0	21	144	503
7.	*C*. *palpifer*	2	121	0	0	26	149	2	118	0	0	18	138	287
8.	*C*. *huffi*	3	66	0	0	14	83	2	105	0	0	12	119	202
9.	*C*. *shortti*	1	86	0	0	9	96	1	48	0	0	17	66	162
10.	*C*. *guttifer*	2	93	3	0	17	115	2	49	3	0	26	80	195
11.	*C*. *fulvus*	7	49	0	0	57	113	1	60	0	0	14	75	188
12.	*C*. *orientalis*	1	22	0	0	48	71	0	0	0	0	0	0	71
13.	*C*. *fordae*	0	0	0	0	0	0	0	138	0	0	16	154	154
14.	*C*. *insignipennis*	0	0	0	0	0	0	0	11	0	0	0	11	11
15.	*Culicoides* sp. (unknown)	0	30	2	5	0	37	0	0	0	0	0	0	37
	Total	119	2,983	19	8	700	3,829	51	2,292	10	4	392	2,749	6,578

### Parasite infection status in the wild-caught biting midges

**Figure 3:**
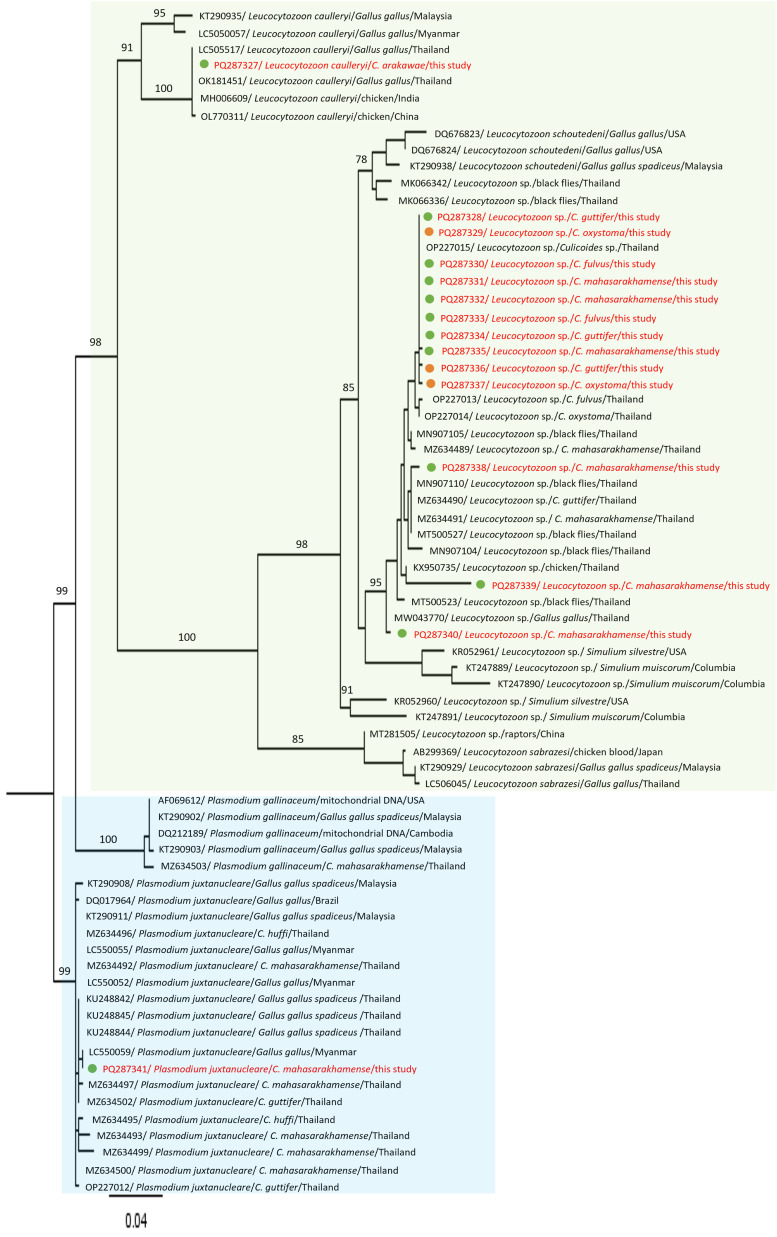
Phylogenetic construction involving 15 sequences from the current study and 56 reference sequences (23 sequences of *Plasmodium* species and 33 sequences of *Leucocytozoon* species) retrieved from GenBank. These sequences were analyzed using the maximum likelihood method with the TIM+F+G4 model under 1,000 bootstrap replicates using IQTree software. Tree with branch lengths indicating number of substitutions per site. The sequences from the present study are written in red, whereas the green-dot circles refer to samples from Chiang Mai province (northern) and orange-dot circles refer to samples from Nakhon Si Thammarat province (southern), Thailand.

A total of 738 biting midges (parous) were used to screen for parasite infection status. As per the results, no genomic DNA samples of *Leishmania* spp., trypanosomatids, and filarial nematodes were detected. Fifteen haemosporidian mitochondrial cyt *b* sequences from five species of biting midges, *i.e.*, *C*. *arakawae* (6.7%), *C*. *mahasarakhamense* (46.7%), *C*. *oxystoma* (13.3%), *C*. *guttifer* (20%), and *C*. *fulvus* (13.3%) were investigated. The DNA of *Leucocytozoon* spp., *Le. caulleryi*, and *P. juxtanucleare* parasites was detected in 13, 1, and 1 *Culicoides* sample(s), respectively. Identified parasite species in selected *Culicoides* biting midges (parous) collected from Chiang Mai and Nakhon Si Thammarat provinces are shown in Table 4. Resulting haemosporidian sequences were determined for sequence similarity compared to other global sequences. One sequence produced here (accession no. PQ287341) showed high similarity (100.00%) with one sequence identified as *P. juxtanucleare* (accession No. LC550059), another (accession No. PQ287327) showed 99.81% similarity with one sequence identified as *Le. caulleryi* (accession No. OK181451), whereas 13 other sequences (accession Nos. PQ287328–PQ287340) showed similarities (95.90% to 100.00%) with sequences identified at the genus level, namely *Leucocytozoon* (Fig. 3).

**Table 4:** Molecular identification of parasites in selected *Culicoides* biting midges (Parous) collected from Chiang Mai and Nakhon Si Thammarat provinces, Thailand, February–July 2024.

*Culicoides* species	Number of biting midges								Parasite species (insect code)
	Chiang Mai				Nakhon Si Thammarat				
	SS	DS	HD	Total	NB	NT	TS	Total	
*C*. *asiana*	32 (5, 0)^1^	22 (5, 0)	38 (10, 0)	92 (20, 0)	12 (5, 0)	5 (5, 0)	9 (5, 0)	26 (15, 0)	
*C*. *peregrinus*	215 (20, 0)	198 (18, 0)	124 (12, 0)	537 (50, 0)	163 (10, 0)	206 (20, 0)	236 (20, 0)	605 (50, 0)	
*C*. *innoxius*	223 (20, 0)	135 (10, 0)	123 (10, 0)	481 (40, 0)	282 (20, 0)	169 (15, 0)	196 (20, 0)	647 (55, 0)	
*C*. *arakawae*	238 (20, 1)	128 (10, 0)	195 (20, 0)	561 (50, 1)	108 (10, 0)	65 (10, 0)	53 (10, 0)	226 (30, 0)	*Leucocytozoon caulleryi* (SS15)
*C*. *mahasarakhamense*	248 (30, 4)	142 (20, 3)	191 (20, 0)	581 (70, 7)	59 (10, 0)	45 (10, 0)	33 (10, 0)	137 (30, 0)	*Leucocytozoon* sp*.* (SS1, SS9, SS30, DS7, DS8, DS9)
									*Plasmodium juxtanucleare* (SS25)
*C*. *oxystoma*	110 (10, 0)	68 (10, 0)	86 (10, 0)	264 (30, 0)	34 (15, 0)	37 (15, 0)	51 (20, 2)	122 (50, 2)	*Leucocytozoon* sp. (TS4, TS7)
*C*. *palpifer*	51 (5, 0)	35 (5, 0)	35 (5, 0)	121 (15, 0)	48 (5, 0)	32 (5, 0)	38 (5, 0)	118 (15, 0)	
*C*. *huffi*	29 (5, 0)	17 (5, 0)	20 (5, 0)	66 (15, 0)	49 (5, 0)	19 (5, 0)	37 (5, 0)	105 (15, 0)	
*C*. *shortti*	45 (5, 0)	30 (5, 0)	11 (5, 0)	86 (15, 0)	27 (5, 0)	14 (5, 0)	7 (5, 0)	48 (15, 0)	
*C*. *guttifer*	40 (10, 0)	19 (10, 2)	34 (10, 0)	93 (30, 2)	3 (2, 0)	18 (10, 0)	28 (15, 1)	49 (27, 1)	*Leucocytozoon* sp. (DS3, DS4, TS1)
*C*. *fulvus*	6 (4, 0)	15 (12, 2)	28 (13, 0)	49 (29, 2)	9 (8, 0)	28 (16, 0)	23 (9, 0)	60 (33, 0)	*Leucocytozoon* sp. (DS5, DS6)
*C*. *orientalis*	13 (5, 0)	2 (2, 0)	7 (5, 0)	22 (12, 0)	0	0	0	0	
*C*. *fordae*	0	0	0	0	45 (5, 0)	67 (7, 0)	26 (5, 0)	138 (17, 0)	
*C*. *insignipennis*	0	0	0	0	4 (2, 0)	7 (3, 0)	0	11 (5, 0)	
*Culicoides* sp. (unknown)	0	0	30 (5, 0)	30 (5, 0)	0	0	0	0	
Total	1,250	811	922	2,983	843	712	737	2,292	

Abbreviations: San Sai (SS); Doi Saket (DS); Hang Dong (HD); Na Bon (NB); Nopphitam (NT); Tha Sala (TS).

1 The numbers inside the brackets represent the number of dissected parous insects (front) and the number of parous insects that were found to be positive for parasite (behind).

### Host preference of the biting midges

**Figure 4:**
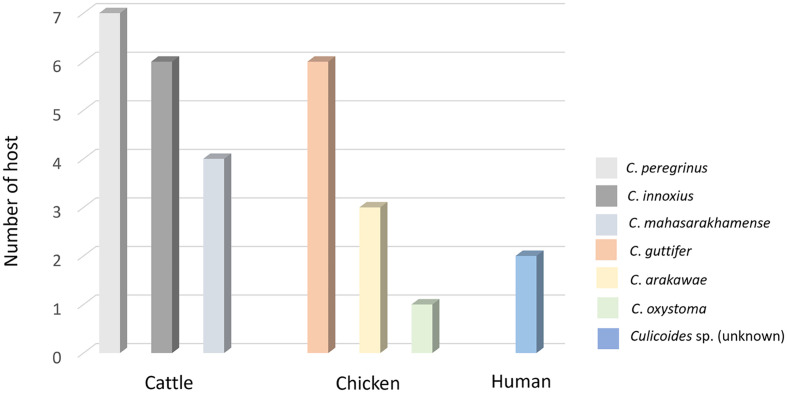
Host preference analysis of 29 engorged females of seven different *Culicoides* species.

Host blood meal identification was conducted in a total of 29 engorged *Culicoides* biting midges, including *C*. *peregrinus* (*n* = 7), *C*. *innoxius* (*n* = 6), *C*. *guttifer* (*n* = 6), *C*. *mahasarakhamense* (*n* = 4), *C*. *arakawae* (*n* = 3), *C*. *oxystoma* (*n* = 1), and unknown *Culicoides* sp. (*n* = 2; accession Nos. PQ287342–PQ287343) (Table 3). Three different vertebrate hosts, *i.e.*, cattle (*Bos indicus*), chickens (*Gallus gallus*), and humans (*Homo sapiens*), were determined as preference hosts. In the study areas, *C*. *peregrinus*, *C*. *innoxius*, and *C*. *mahasarakhamense* fed on cattle with 58.6% (17/29) (*n* = 17; accession Nos. PQ287344–PQ287360), whereas *C*. *arakawae*, *C*. *oxystoma*, and *C*. *guttifer* were found to feed on chickens with 34.5% (10/29) (*n* = 10; accession Nos. PQ287361–PQ287370). Interestingly, we found two unknown *Culicoides* sp. that fed on humans with 6.9% (2/29) (*n* = 2; accession Nos. PQ287371–PQ287372). None of the samples with parasite DNA showed coinfection, and no *Culicoides* specimens were detected for mixed-host blood meal DNA. The relationships of the investigated blood meal remnants in seven *Culicoides* species are shown in Figure 4.

## Discussion

This study provides information about the most dominant biting midge species in selected mixed-livestock farming areas in Chiang Mai and Nakhon Si Thammarat provinces, Thailand. In addition, the molecular occurrence of parasites in five *Culicoides* species is shown, and the host preference of wild-caught biting midges was determined. In our study, *C*. *arakawae* and *C*. *mahasarakhamense* were investigated as the most dominant *Culicoides* species, followed by *C*. *peregrinus* and *C*. *innoxius* in Chiang Mai, whereas *C*. *innoxius* and *C*. *peregrinus* in Nakhon Si Thammarat province. So far, *C*. *mahasarakhamense*, *C*. *peregrinus*, and *C*. *innoxius* have been investigated as the most dominant *Culicoides* in different areas of eastern, northern, and southern Thailand [14, 39, 40, 53, 54]. Therefore, our study revealed common findings of dominant *Culicoides* species similar to the above-mentioned studies. Three species of *Culicoides*, including *C*. *mahasarakhamense*, *C*. *peregrinus*, and *C*. *innoxius* are expected to be widely distributed and are probably the most dominant *Culicoides* species in Thailand, suggesting that the local transmission capabilities of the biting midges for vector-borne pathogens should be considered. In addition, all the *Culicoides* samples were collected near mixed-livestock farms with a particular distance (10–30 m) from the farm owner’s house in the current study. Therefore, these dominant species appeared to be capable of pathogen transmission in nature as well [7].

In our samples, no genomic DNA samples of *Leishmania* spp., trypanosomatids, and filarial nematodes were detected. This may be due to variations in insect species and sampling regions. Despite some *Culicoides* species facilitating parasite development in laboratory studies, this may not occur under natural conditions where transmission dynamics are influenced by ecological and host-related factors [54]. In our study, DNA of haemosporidians, *Leucocytozoon* sp., and *P*. *juxtanucleare* was detected in *C*. *mahasarakhamense*, *C*. *oxystoma*, *C*. *guttifer*, and *C*. *fulvus*, which is similar to previous studies in Thailand [35, 39, 46, 53]. Interestingly, *Le*. *caulleryi* DNA was detected for the first time in Thailand inside the biting midge *C*. *arakawae* in the present study. This is consistent with a previous study on the experimental infection of *C*. *arakawae* by *Le*. *caulleryi* to prove that the insect species is a vector of this parasite [60]. In addition to this, *Le*. *caulleryi* has been diagnosed by histopathology and molecular examination in chickens in South Korea, as the causal agent of chicken leucocytozoonosis [29]. Therefore, we should be aware of *Le*. *caulleryi* infection in chickens in Thailand. Further studies on the prevalence of *Le*. *caulleryi* infection in chickens in Thailand should be carried out, and the role of *C*. *arakawae* as a natural vector of this parasite should be investigated through the presence of the parasite inside this vector. Although *Culex* and *Ochlerotatus* mosquitoes have been described as avian *Plasmodium* vectors [12], the DNA of *P*. *juxtanucleare* has been detected in *C*. *mahasarakhamense*, as reported by Pramual *et al.* [39]. Therefore, the role of *C*. *mahasarakhamense* as a vector of *Plasmodium* species in Thailand should be further investigated.

According to the phylogenetic analyses, four unnamed *Leucocytozoon* sp. detected in the current study were genetically related to *Leucocytozoon* sp. found in biting midges (*Culicoides* sp., *C*. *fulvus*, *C*. *oxystoma*, *C*. *mahasarakhamense*, and *C*. *guttifer*), chickens (*Gallus gallus*), and black flies (*Simulium* spp.) previously reported in Thailand [21, 38, 41, 53]. In addition, a *Le*. *caulleryi* sequence detected in the present study showed a strong genetic relationship with *Le*. *caulleryi* extracted from chickens (*Gallus gallus*) in Thailand [9]. Moreover, *P*. *juxtanucleare* detected in *C*. *mahasarakhamense* from our study belonged to a clade of *P*. *juxtanucleare* that has been reported to infect chickens (*Gallus gallus*) and biting midges of *C*. *mahasarakhamense*, *C*. *guttifer*, and *C*. *huffi* in Thailand and Myanmar [39, 53, 55, 58]. Further experimental investigation is needed to demonstrate the role of biting midges as competence vectors of the above haemosporidian parasites.

Determining host blood DNA in arthropod vectors is crucial to better understand the transmission of vector-borne pathogens. Globally, molecular identification of blood meals in *Culicoides* biting midges has been done in Germany [2], Spain [32], France [15], Tunisia [49], Serbia [57], Romania [56], India [25], and the United States [33]. According to the results, *C*. *peregrinus*, *C*. *innoxius*, and *C*. *mahasarakhamense* preferred to feed on cattle, whereas *C*. *arakawae*, *C*. *oxystoma*, and *C*. *guttifer* were found to feed on chickens. *Culicoides peregrinus* and *C*. *innoxius* are known to primarily feed either on cattle or on at least two mammalian hosts, such as cattle and sheep [20, 25]. However, a *C*. *mahasarakhamense* with a cattle blood meal was detected for the first time in this study. This may be caused by opportunistic feeding, as *C. mahasarakhamense* has been reported feeding on chickens, and avian parasites have been identified within it [39]. The presence of a cattle blood meal in *C*. *mahasarakhamense* underscores the need for further investigations of their feeding behavior and host specificity. Furthermore, *C*. *arakawae*, *C*. *oxystoma*, and *C*. *guttifer* were found to feed on chickens rather than other hosts. Similarly, *C*. *arakawae* is thought to be a common pest on poultry farms, where they primarily feed on chicken blood [20]. In addition, *C*. *oxystoma* is reported to show host preference in a wide range of vertebrates [23]. To the best of our knowledge, this is the first study detecting chicken DNA in *C*. *oxystoma* in Thailand. Previously, chicken DNA has been detected in several *Culicoides* species, including *C*. *guttifer* in Thailand [14, 20, 54]. In some parts of Thailand, *C*. *oxystoma*, *C*. *imicola*, *C*. *brevitarsis*, *C*. *peregrinus*, and *C*. *guttifer* have been reported with canine DNA [23, 53], even though canines are considered a less preferred host for biting midges. However, no canine DNA was detected in this study.

Although cattle and chickens have been considered major sources of feeding for biting midges, recently, human DNA has been found in *C*. *oxystoma*, *C*. *imicola*, and *C*. *brevitarsis* in Thailand [14, 23]. Interestingly, in this study, two unknown *Culicoides* sp. were found to feed on humans, suggesting that more anthropophilic *Culicoides* species remain to be discovered. Importantly, anthropophilic *Culicoides* species are likely to inhabit nearby human residences with year-round banana and plantain cultivation, since this environment provides a highly favourable setting for their development [10]. In the current study, the trapping locations were close to residential areas (10–30 m), making it easy for the insects to feed on the available hosts, *i.e.*, cattle (6–15) and chickens (>20– > 60 in some places). However, the study failed to detect any canine DNA. This may be due to the small number of dogs at the sampling sites. Apart from the influence of host availability near the traps, host-switching behavior in *Culicoides* biting midges can occur if feeding is interrupted, which may play a significant role in their capacity for pathogen transmission [37, 53]. Environmental factors such as temperature, humidity, and the presence of specific vegetation can alter the availability and attractiveness of hosts [54].

## Conclusion

Overall, our study provides current knowledge on the most abundant *Culicoides* biting midges in Chiang Mai and Nakhon Si Thammarat provinces. Out of 15 species, *C*. *mahasarakhamense*, *C*. *arakawae*, *C*. *oxystoma*, *C*. *guttifer*, and *C*. *fulvu*s might be potential vectors of haemosporidians. Three vertebrate host blood DNA types (cattle, chicken, and humans) were detected from seven *Culicoides* species. Nonetheless, our study highlighted that many *Culicoides* species remain to be investigated, and cattle are at risk in mixed-livestock farming practices, as they are the targeted hosts of biting midges. Until now, parasitic diseases transmitted by *Culicoides* biting midges have been overlooked. Understanding the habitual actions of these insects in accepting different geographical regions could be beneficial, and more surveillance research should be performed.

## Supplementary material

The supplementary material for this article can be found at https://www.parasite-journal.org/10.1051/parasite/2024082/olm.
